# Promoting HIV indicator condition-guided testing in hospital settings (PROTEST 2.0): study protocol for a multicentre interventional study

**DOI:** 10.1186/s12879-021-06183-8

**Published:** 2021-06-02

**Authors:** Saskia J. Bogers, Maarten F. Schim van der Loeff, Udi Davidovich, Anders Boyd, Marc van der Valk, Kees Brinkman, Godelieve J. de Bree, Peter Reiss, Jan E. A. M. van Bergen, Suzanne E. Geerlings, on behalf of the HIV Transmission Elimination AMsterdam (H-TEAM) Consortium, J. E. A. M. van Bergen, P. Brokx, F. Deug, M. Heidenrijk, M. Prins, P. Reiss, M. van der Valk, G. J. de Bree, P. Brokx, U. Davidovich, S. E. Geerlings, E. Hoornenborg, A. Oomen, A. van Sighem, W. Zuilhof, N. Schat, R. C. A. Achterbergh, M. van Agtmael, J. Ananworanich, D. Van de Beek, G. E. L. van den Berk, D. Bezemer, A. van Bijnen, W. L. Blok, S. Bogers, M. Bomers, C. A. B. Boucher, W. Brokking, D. Burger, K. Brinkman, N. Brinkman, M. de Bruin, S. Bruisten, L. Coyer, R. van Crevel, C. G. Daans, L. Dellemann, M. Dijkstra, Y. T. van Duijnhoven, A. van Eeden, L. Elsenburg, M. A. M. van den Elshout, C. Ester, E. Ersan, P. E. V. Felipa, P. H. J. Frissen, T. B. H. Geijtenbeek, M. H. Godfried, J. van Gool, A. Goorhuis, M. Groot, C. A. Hankins, A. Heijnen, M. M. J. Hillebregt, A. Hogewoning, M. Hommenga, J. W. Hovius, Y. Janssen, K. de Jong, V. Jongen, N. A. Kootstra, R. A. Koup, F. P. Kroon, T. J. W. van de Laar, F. Lauw, M. M. van Leeuwen, K. Lettinga, I. Linde, D. S. E. Loomans, J. T. van der Meer, T. Mouhebati, B. J. Mulder, J. Mulder, F. J. Nellen, A. Nijsters, H. Nobel, P. Oostvogel, E. L. M. Op de Coul, E. Peters, I. S. Peters, T. van der Poll, O. Ratmann, C. Rokx, M. S. van Rooijen, M. F. Schim van der Loeff, W. E. M. Schouten, G. J. Sonder, J. Veenstra, A. Verbon, F. Verdult, J. de Vocht, H. J. de Vries, S. Vrouenraets, M. van Vugt, W. J. Wiersinga, F. W. Wit, L. R. Woittiez, S. Zaheri, P. Zantkuijl, M. C. van Zelm, A. Żakowicz, H. M. L. Zimmermann

**Affiliations:** 1 Department of Internal Medicine, Division of Infectious Diseases, Amsterdam University Medical Centers, University of Amsterdam, room D3-226, Meibergdreef 9, 1105 AZ Amsterdam, the Netherlands; 2 Department of Infectious Diseases, Public Health Service of Amsterdam, Amsterdam, the Netherlands; 3 HIV Monitoring Foundation, Amsterdam, the Netherlands; 4 Department of Internal Medicine, Onze Lieve Vrouwe Gasthuis, Amsterdam, the Netherlands; 5 Amsterdam Institute for Global Health and Development, Amsterdam, the Netherlands; 6 Department of Global Health, Amsterdam University Medical Centers, University of Amsterdam, Amsterdam, the Netherlands; 7 Department of General Practice, Amsterdam University Medical Centers, location Academic Medical Center, University of Amsterdam, Amsterdam, the Netherlands; 8 STI AIDS Netherlands, Amsterdam, the Netherlands

**Keywords:** Indicator condition, HIV testing, Healthcare quality improvement, Implementation, Multifaceted intervention

## Abstract

**Background:**

Late presentation remains a key barrier towards controlling the HIV epidemic. Indicator conditions (ICs) are those that are AIDS-defining, associated with a prevalence of undiagnosed HIV > 0.1%, or whose clinical management would be impeded if an HIV infection were undiagnosed. IC-guided HIV testing is an effective strategy in identifying undiagnosed HIV, but opportunities for earlier HIV diagnosis through IC-guided testing are being missed. We present a protocol for an interventional study to improve awareness of IC-guided testing and increase HIV testing in patients presenting with ICs in a hospital setting.

**Methods:**

We designed a multicentre interventional study to be implemented at five hospitals in the region of Amsterdam, the Netherlands. Seven ICs were selected for which HIV test ratios (proportion of patients with an IC tested for HIV) will be measured: tuberculosis, cervical/vulvar cancer or high-grade cervical/vulvar dysplasia, malignant lymphoma, hepatitis B and C, and peripheral neuropathy. Prior to the intervention, a baseline assessment of HIV test ratios across ICs will be performed in eligible patients (IC diagnosed January 2015 through May 2020, ≥18 years, not known HIV positive) and an assessment of barriers and facilitators for HIV testing amongst relevant specialties will be conducted using qualitative (interviews) and quantitative methods (questionnaires). The intervention phase will consist of an educational intervention, including presentation of baseline results as competitive graphical audit and feedback combined with discussion on implementation and opportunities for improvement. The effect of the intervention will be assessed by comparing HIV test ratios of the pre-intervention and post-intervention periods. The primary endpoint is the HIV test ratio within ±3 months of IC diagnosis. Secondary endpoints are the HIV test ratio within ±6 months of diagnosis, ratio ever tested for HIV, HIV positivity percentage, proportion of late presenters and proportion with known HIV status prior to initiating treatment for their IC.

**Discussion:**

This protocol presents a strategy aimed at increasing awareness of the benefits of IC-guided testing and increasing HIV testing in patients presenting with ICs in hospital settings to identify undiagnosed HIV in Amsterdam, the Netherlands.

**Trial registration:**

Dutch trial registry: NL7521. Registered 14 February 2019.

**Supplementary Information:**

The online version contains supplementary material available at 10.1186/s12879-021-06183-8.

## Background

In our efforts to complete the ‘last mile’ towards ending the HIV epidemic, timely diagnosis of HIV remains a key focal point. Globally, about 19% of the estimated 38.0 million people living with HIV (PLHIV) were unaware of their HIV status in 2019 [1]. In Europe and Central Asia, one in five PLHIV remain undiagnosed and half of new diagnoses in the European Union are at a late stage of infection (CD4 count < 350 cells/mm^3^ or presenting with an AIDS-defining event) [2]. These figures are worrisome as late presentation is associated with higher morbidity and mortality, poorer response to combination antiretroviral therapy (cART) and onward transmission of HIV [3–6].

One of the strategies to improve timely HIV diagnosis is testing for HIV in all patients diagnosed with an indicator condition (IC). ICs are defined as conditions that are AIDS-defining, that are associated with a prevalence of undiagnosed HIV > 0.1% (the threshold for cost-effectiveness in HIV testing [7, 8]), or whose clinical management would be adversely affected if HIV infection were not identified. The HIV Indicator Diseases across Europe Study (HIDES) and the subsequent HIDES II study [9, 10] identified various ICs associated with an HIV prevalence of over 0.1%. Currently, over 50 ICs for which HIV testing is recommended are recognised and numerous studies have shown that IC-guided HIV testing is an effective approach to identify undiagnosed PLHIV [11–15]. IC-guided testing also has the advantage that discussing patient risk factors for HIV, which still poses a barrier for some physicians [16, 17], can be bypassed. As a result, HIV testing and care guidelines across Europe have now recommended IC-guided testing [18]. However, various studies have recently shown low adherence to these recommendations. Although they confirmed a prevalence of undiagnosed HIV > 0.1% amongst patients diagnosed with ICs or, conversely, a high prevalence of ICs amongst newly diagnosed PLHIV, there were consistently low HIV testing ratios in patients presenting with ICs and thus missed opportunities for earlier HIV diagnosis [19–24]. Furthermore, in 2017, the majority of specialty guidelines for ICs did not recommend HIV testing, making awareness amongst medical specialties other than those actively involved in HIV care less likely [25].

In the Netherlands, an estimated 8% of PLHIV is unaware of their diagnosis and over half of all newly diagnosed cases involve late presentation [26]. In the hospital setting, 69% of new HIV diagnoses are late-stage [26]. Previous research has shown that there have been missed opportunities to identify undiagnosed PLHIV through IC-guided testing [14]. As an estimated 27% of PLHIV in the Netherlands live in Amsterdam and over one in five HIV diagnoses are made there, a city-based approach to curb the Dutch HIV epidemic is essential. This led to the establishment of the HIV Transmission Elimination Amsterdam (H-TEAM) consortium in 2014. It deploys a city-based combination intervention strategy focussing on all parts of the HIV prevention and care cascade [27]. The H-TEAM designed an interventional study to promote IC-guided HIV testing at hospitals in the region of Amsterdam. Here, we describe the details of a protocol for an interventional study to (1) generate awareness about ICs and the importance of IC-guided HIV testing amongst physicians working in hospitals, and (2) improve the HIV test ratio in ICs amongst different medical specialties in the region of Amsterdam, the Netherlands.

## Methods

### Setting and study design

We designed a multicentre interventional study that will take place at 5 hospitals (two university hospitals, two non-academic teaching hospitals and one non-teaching hospital) in the region of Amsterdam, the Netherlands.

The development of the study consisted of three phases; an elicitation phase, a design phase, and an implementation phase. The elicitation and design phases have already been completed, while the implementation phase is currently taking place (as of June 2020). During the elicitation phase, a group of four experts, including an infectious diseases physician, a general practitioner, an epidemiologist, and a behavioural scientist, identified essential elements for an empirically based intervention. Additionally, two infectious diseases physicians from two participating hospitals were consulted on the perceived feasibility of the identified elements. During the design phase, the study protocol and evaluation plan were composed, with additional consultation from an expert on methodological approaches and statistical analysis. During the implementation phase, we will implement the educational intervention and assess its impact. The pre-intervention HIV test ratio (i.e. the proportion of patients presenting with an IC who are tested for HIV) will be compared to the post-intervention test ratio for seven selected ICs. To this end, pre-intervention test data over a period of 5.5 years (January 2015 through May 2020) will be retrospectively collected. As all participating hospitals have utilised their current electronic health record (EHR) software from 2015, allowing readily available data, the starting point of the pre-intervention period was selected at that year. The intervention period will last 6 months and its effect will be evaluated for a period of 1 year from the start of the intervention (June 2020 through May 2021, Fig. 1).

**Fig. 1:**
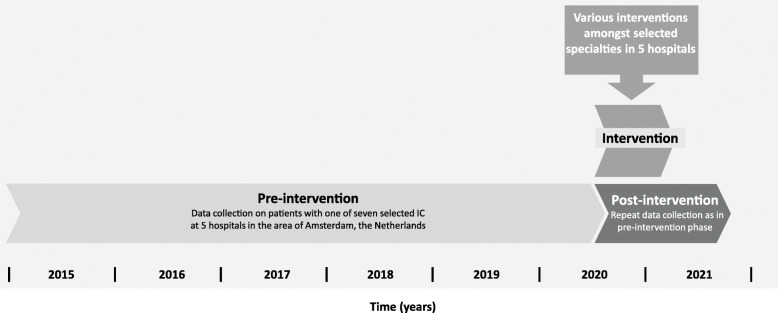
Intervention design to promote IC-guided testing for HIV. IC = indicator condition

### Selection of indicator conditions

Seven ICs were selected for inclusion in this study: tuberculosis (TB), cervical cancer or high-grade cervical dysplasia, vulvar cancer or high-grade vulvar dysplasia, malignant lymphoma, hepatitis B (HBV), hepatitis C (HCV) and peripheral neuropathy (Table 1). These ICs were selected as they are managed by several different medical specialties (pulmonology, gynaecology, haematology, gastroenterology and neurology) and their evidence of being associated with HIV is variable. For example, the association of HIV and TB has been extensively documented, but less evidence on the association between HIV and peripheral neuropathy is available. We additionally selected these ICs based on their relatively high incidence, based on reports by the various specialty associations.

**Table 1:** Selected indicator conditions, associated specialties, and specific selection criteria for eligibility

Indicator condition	Main Specialty	Indicator condition-specific selection criteria
Tuberculosis	Pulmonology	Patients with latent *M. tuberculosis* infection, but no tuberculosis disease, are excluded
Cervical cancer or CIN 3+	Gynaecology	Patients without biopsy-confirmed high-grade dysplasia (CIN 3+) or carcinoma (invasive or non-invasive) are excluded
Vulvar cancer or VIN 3+	Gynaecology	Patients without biopsy-confirmed high-grade dysplasia (VIN 3+) or carcinoma (invasive or non-invasive) are excluded
Malignant lymphoma	Haematology	All types of malignant lymphoma, including all subtypes of Hodgkin’s lymphoma and non-Hodgkin lymphoma are included
Hepatitis B	Gastroenterology	Both acute and chronic hepatitis B cases are included
Hepatitis C	Gastroenterology	Both acute and chronic hepatitis C cases are included
Peripheral neuropathy	Neurology	Patients with known diabetes mellitus before presenting and patients for whom no diagnostic laboratory workup was indicated are excluded

*CIN* cervical intraepithelial neoplasia, *VIN* vulvar intraepithelial neoplasia

### Patient selection and inclusion

**Fig. 2:**
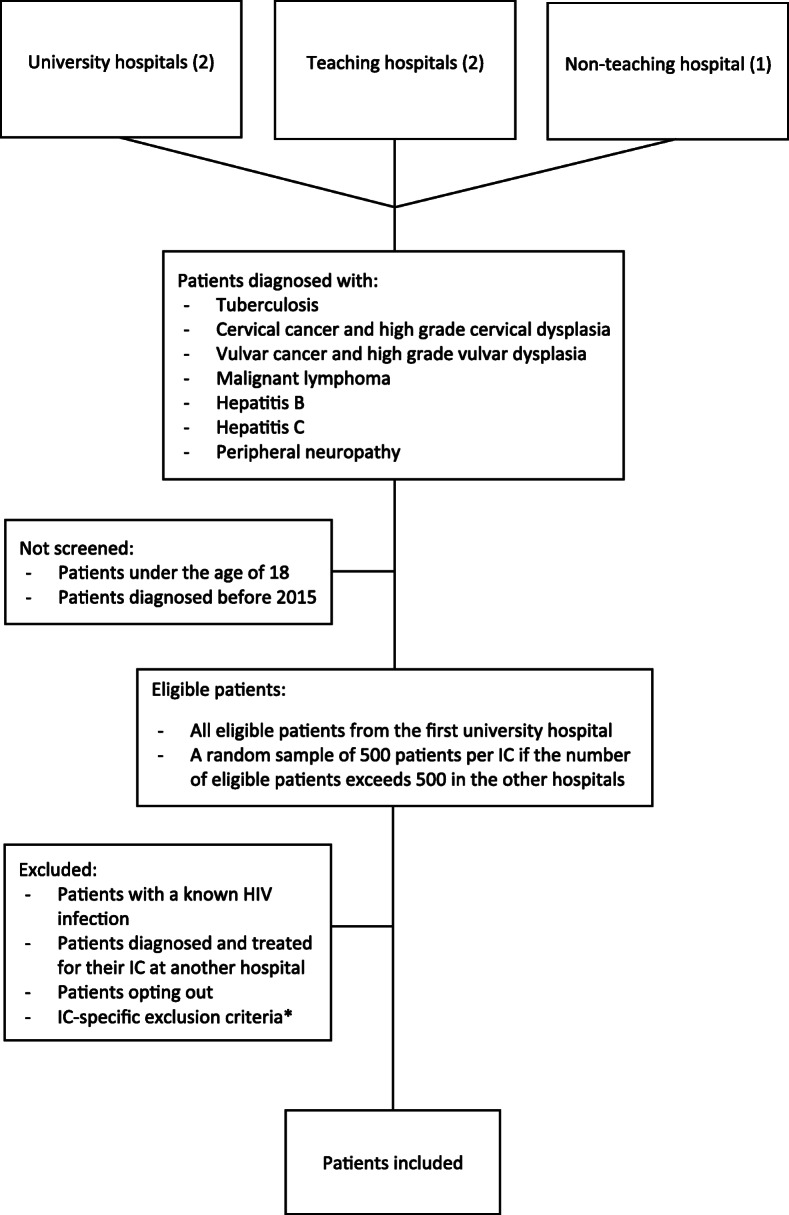
Flowchart of patient inclusion. * See Table 1 for an overview. IC = indicator condition

The HIV testing ratio will be assessed using patient data from EHRs. Eligible patients will be identified using national disease billing codes. Patients of ≥18 years, diagnosed with one of the selected ICs will be eligible for inclusion (Fig. 2). The following patients will be excluded: (1) patients with a known HIV infection prior to presenting with the selected IC and (2) patients that are diagnosed and treated for their IC at another hospital, and the relevant billing code was only recorded in the EHR for administrative purposes. Patients who are referred by another physician for a second opinion or transferred from another hospital will be included. IC-specific selection criteria will additionally be applied (Table 1). In both the pre- and post-intervention period, all eligible patients from the first university hospital will be included. For the other four hospitals, data from all eligible patients will be included if there are ≤500 patients per IC; while data from a random sample of 500 patients will be assessed for eligibility and, if eligible, included if the number of patients per IC exceeds 500. This was done to keep workload manageable as the added precision of inclusions > 500 is limited. Eligible patients will be given the opportunity to opt-out of the use of their EHR data.

### Assessments

For all included patients, year of birth, sex, socio-economic status (SES; as derived from the postal code of residence) and whether deceased (including date of death) will be recorded. For women, any pregnancy at the time of IC diagnosis will be recorded. Additionally, the date of diagnosis and, if applicable, treatment of the specific IC will be recorded. To determine outcome measures, if, when and where an HIV test was performed will be recorded. To this end, all laboratory results, scanned documents, patient communication and referral letters in the EHR will be searched. All female patients with an EHR-recorded pregnancy after January 2004 will be assumed to have been tested for HIV during pregnancy, as all pregnant women in the Netherlands are tested for HIV on an opt-out basis, as part of the antenatal care programme, and the number opting out is negligible [28]. When no HIV test was performed during the diagnostic work-up for the IC, the EHR will be searched to assess whether a reason was given by the healthcare provider for not offering an HIV test or by the patient for declining the test. If the result of an HIV-test was positive, we will record the CD4 count at diagnosis. Electronic Case Report Forms (eCRFs) will be used for data collection using Castor (Castor Electronic Data Capture, Amsterdam, the Netherlands). As a quality control check, 10 % of eCRFs per IC and hospital will be randomly selected and verified by a second researcher. If the discrepancy in findings is > 2.5%, all eCRFs for that IC and hospital will be verified by the second researcher.

### Endpoints

The primary endpoint is the HIV test ratio (i.e. the proportion of patients with an IC who were tested for HIV) within ±3 months of IC diagnosis (3 months before to 3 months after diagnosis). Secondary endpoints are the HIV test ratio within ±6 months of IC diagnosis; proportion of patients presenting with an IC that were ever tested for HIV; HIV positivity ratio (i.e. number of positive HIV tests of the total number of HIV tests performed), both for the ±3 month and ± 6 month window; proportion of new HIV diagnoses that are late-stage (defined as having a CD4 count < 350 cells/mm^3^ at diagnosis); and proportion of patients tested for HIV before initiating treatment for their IC.

### Intervention strategy

A multifaceted intervention strategy will be used to improve HIV testing at different medical specialties who primarily diagnose and treat patients with the selected ICs (Table 2). The interventions will be tailored to specialty-specific circumstances. Opportunities for intervention will be identified through qualitative and quantitative research, specifically by addressing barriers for IC-guided testing amongst professionals and their work settings [29, 30]. First, an overview of IC-guided HIV testing recommendations will be made from the local and national specialty guidelines of the selected ICs. For relevant guidelines without such recommendations, the possibility to amend these guidelines will be explored. Second, all medical specialists and residents from each involved specialty at the participating hospitals will be invited to complete an online questionnaire on barriers and facilitators for HIV testing in patients with ICs related to their discipline. To this end, a questionnaire was developed based on the Attitude-Social norm-self Efficacy (ASE) model [31, 32], which is an evidence-based instrument to assess behavior and its determinants in healthcare personnel (Supplementary appendix 1). The questionnaire will be distributed via email by contact persons representing each specialty at each hospital. The proportion responding will be calculated as the number of respondents divided by the number of recipients. Third, attitudes towards IC-guided HIV testing, and opportunities for improvement that fit the respondent’s specialty and hospital, will be assessed through semi-structured interviews with medical specialists and residents. Individuals who respond to the online questionnaire and are willing to provide contact information will be recruited for these interviews. The contact persons of the various specialties will also be invited to participate in the interviews. The outcomes and opportunities for improvement from these interviews will be used for the educational intervention meetings (Supplementary appendix 2).

**Table 2:** Planned study components to promote indicator condition-guided testing for HIV

**Pre-intervention**	
Assess recommendations for HIV testing in local and national IC specialty guidelines (literature review)	
**Intervention**	
Map barriers and facilitators to IC-guided HIV testing (online questionnaire)	
Assessing specialty specific opportunities to optimise HIV testing practices (semi-structured interviews)	
Educational meeting for medical specialists and residents on IC-guided HIV testing (presentation)	
Interactive discussion on opportunities to optimise HIV testing practices	
Competitive feedback on IC-guided HIV testing performance (graphical audit and feedback)	
Education material (pocket cards and posters)	
**Post-intervention**	
Reporting of post-intervention feedback on IC-guided HIV testing performance to participating specialties (graphical audit and feedback)	

*IC* indicator condition

We will offer to host an educational meeting of about 30–45 min for each medical specialty at each participating hospital. During these educational meetings, the current state of the HIV epidemic in the Netherlands, evidence on the relation between HIV and the relevant ICs and evidence on IC-guided testing for HIV will first be presented. In addition to the ICs selected for this study, the entire list of currently recognised ICs will be presented with the aim of highlighting other ICs relevant to the specialty and bringing a more comprehensive awareness of IC-guided testing for HIV. Baseline HIV test ratios for all hospitals will be presented. This technique is known as competitive graphical audit and feedback, which was chosen because of its effectiveness in improving guideline adherence [33]. Finally, the results of the questionnaires and interviews, and identified barriers and opportunities for improvement, will be presented, followed by an interactive discussion on opportunities to improve HIV testing. When suggestions for improvement strategies are made by the participants during this discussion or other phases of the implementation period, we will offer assistance in implementation. At the end of the meeting, educational materials (pocket cards and posters) will be handed out to remind participants of the topics discussed. Participants will be informed that the effect of the intervention will be assessed through a post-intervention assessment of the HIV test ratio and that these results will be reported back to all participating hospitals.

### Statistical analysis

The number of patients with ICs will be reported per IC, per hospital, and per period (in the pre- or post-intervention periods) as well as the number and percentage of patients with an IC who were tested for HIV within ±3 months of IC diagnosis. Additionally, the number and percentage of patients with an IC who were tested for HIV within ±6 months of IC diagnosis and the number and percentage of patients with an IC who were ever tested for HIV will be reported.

A time-series approach using segmented, Poisson regression will be used to evaluate the effect of the intervention. We will first model the HIV test ratio as a function of calendar time (in quarter-year periods), intervention period (pre- versus post-intervention), and the interaction between the two. If the interaction term in the model is significant (i.e. differences in slopes), the effect of the intervention will be determined by testing the parameter estimate of the interaction term. If the interaction term is non-significant (i.e. no difference in slopes), the interaction term of the model above will be removed and the effect of the intervention will be tested by the intervention term. Assuming no difference in slopes, average recruitment rate of 31 patients/IC per quarter, an increase in testing from 60 to 80% for four ICs (TB, HBV, HCV, malignant lymphoma) and from 12 to 30% for the three remaining ICs (cervical cancer/high-grade dysplasia, vulvar cancer/high-grade dysplasia, peripheral neuropathy) in the pre- versus post-intervention periods, respectively (unpublished data), there will be > 95% power to determine a difference between intervention arms based on a simulation of 2000 runs. Potential confounding variables will also be added to the regression model. *P*-values will be obtained using a Wald *X*^2^ test and a *p*-value of < 0.05 will be considered statistically significant. Subgroup analyses will be performed for each IC and hospital separately, provided that there is ≥1 individual tested during each intervention period within that stratum. Analyses will be performed using Stata (v15.1, StataCorp, USA).

## Discussion

We describe the design of an interventional study that aims to generate awareness amongst hospital-based physicians of the importance of testing for HIV in patients presenting with ICs and to increase the proportion of patients with an IC who are tested for HIV in various medical specialties.

The designed interventional study has several strengths. During the elicitation phase, we identified multiple intervention strategies based on qualitative and quantitative research that can be used simultaneously. This allows us to implement various innovations in healthcare that have been proven successful in other contexts [29, 30]. Likewise, implementing graphical audit and feedback into the educational intervention for this study will hopefully bring about an effective strategy to increase awareness of IC-guided testing for physicians [33, 34].

During the design phase, we selected a wide array of ICs, some of which have been thoroughly established as indicator conditions and already have included HIV testing as part of their specialty guidelines (e.g. TB, HCV), while for others, this has not been the case (e.g. high grade cervical dysplasia and peripheral neuropathy). Additionally, we considered that specialists from the infectious diseases department will have already been attune to HIV testing. Selecting ICs likely to be diagnosed at a broad range of other departments will ensure an intervention that has a much wider reach and thus has increased generalizability.

For the implementation phase, we will use a timeframe of 6 months around diagnosis of an IC (i.e. 3 months before and 3 months after) when calculating the primary endpoint. One previous study used a period of 3 months after IC diagnosis [35], which might inevitably exclude HIV tests performed during the workup leading to an IC diagnosis. Other studies have used 1 month or 6 months as part of their timeframe [13, 36, 37]. Since testing for HIV is not always the first priority after diagnosing an IC, 1 month may be too restrictive. Conversely, allowing up to 6 months between the diagnosis of an IC and an HIV test may be too long to prevent adverse outcomes related to undiagnosed HIV infection, especially when the patient is a late presenter of HIV infection. Nevertheless, we will use the latter cut-off as part of a secondary analysis.

One limitation that will undoubtedly arise when evaluating the effect of our intervention is the lack of control group, as no data from departments or hospitals that are unexposed to the interventions will be collected. However, the Poisson regression model used in this study will include a time component to establish any changes in HIV testing from the moment our interventions are applied. Second, because the study uses data from EHRs, certain patient characteristics, such as ethnicity and sexual risk behaviour, cannot be included, as they are not consistently reported by physicians. Consequently, no adjustment for these possible confounding variables can be made. However, as international guidelines recommend testing for HIV in all patients presenting with an IC, regardless of other patient risk factors, these are considered inessential for this study. Finally, as we will evaluate the effect of our educational intervention by comparing the HIV test ratio over a period of 1 year from the start of the intervention to a baseline assessment, we will be unable to assess whether any effect would be sustainable in the long term.

In conclusion, we have developed a protocol for an empirically based interventional study to create awareness of and improve IC-guided testing in a hospital setting. During the implementation phase, analysis comparing HIV testing before and after its implementation will determine whether this approach is effective in improving IC-guided testing for HIV at hospitals located in the Amsterdam region, with the aim of facilitating earlier identification of PLHIV who currently remain undiagnosed.

## Supplementary Information

**Additional file 1: Supplementary appendix 1.** Online questionnaire. This supplementary appendix provides additional information on the development, distribution and content of the online questionnaire that will be used in this study.

**Additional file 2: Supplementary appendix 2.** Semi-structured interview guide. This supplementary appendix provides additional information on the recruitment and enrolment for the semi-structured interviews, as well as the interview guide that will be used in this study.

## Data Availability

Not applicable.

## Abbreviations

ASE: Attitude - Social norm - self Efficacy
cART: Combination Antiretroviral Therapy
CIN: Cervical Intraepithelial Neoplasia
eCRF: Electronic Case Report Forms
EHR: Electronic Health Record
HBV: Hepatitis B
HCV: Hepatitis C
HIDES: HIV Indicator Diseases across Europe Study
H-TEAM: HIV Transmission Elimination Amsterdam consortium
IC: Indicator Condition
PLHIV: People living with HIV
SES: Socio-Economic Status
TB: Tuberculosis
VIN: Vulvar Intraepithelial Neoplasia
