# Supplementary material for: Promoting HIV indicator condition-guided testing in hospital settings (PROTEST 2.0): study protocol for a multicentre interventional study
Source: BMC Infect Dis. 2021 Jun 2;21:519. doi: 10.1186/s12879-021-06183-8 (PMC8173796; doi:10.1186/s12879-021-06183-8)
Supplement: Supplementary file 1 — Additional file 1: Supplementary appendix 1. Online questionnaire. This supplementary appendix provides additional information on the development, distribution and content of the online questionnaire that will be used in this study. [file 12879_2021_6183_MOESM1_ESM.docx]

**Appendix 1: Online questionnaire**

**Development and distribution**

All medical specialists and residents from each involved specialty at the participating hospitals are invited to complete an online questionnaire on barriers and facilitators for HIV testing of patients with indicator conditions (ICs) in their discipline. To this end, a questionnaire was developed based on the Attitude - Social norm - self Efficacy (ASE) model^1,2^. The questionnaire will be distributed via email by contact persons at each specialty at each hospital. For each specialty, the relevant selected IC was displayed in the questionnaire (e.g. a pulmonologist would only see questions referring to tuberculosis patients, while a neurologist would only see questions referring to peripheral neuropathy).

**Content**

The questionnaire consists of the following chapters:

1. Respondent’s characteristics
2. Attitudes, barriers and facilitators for HIV testing based on the ASE model*
3. Invitation to a subsequent interview

* Questions in chapter 2 are to be answered on a 5-point Likert scale (e.g. completely disagree to completely agree, very unlikely to very likely)

1. de Vries H, Dijkstra M, Kuhlman P. Self-efficacy: the third factor besides attitude and subjective norm as a predictor of behavioural intentions. Health Education Research. 1988;3(3):273-82.

2. Schellart AJM, Steenbeek R, Mulders HPG, Anema JR, Kroneman H, Besseling JJM. Can self-reported disability assessment behaviour of insurance physicians be explained? Applying the ASE model. BMC Public Health. 2011;11(1):576.

**Overview of questions in the questionnaire:**

Chapter 1 - Respondent’s characteristics

1. Respondent’s sex
2. Respondent’s age
3. Respondent’s affiliated specialty/department
4. Respondent’s position (e.g. medical specialist, resident)
5. Respondent’s number of years work experience in the current specialty/department
6. Respondent’s current affiliated type of hospital (e.g. university hospital, teaching hospital)
7. Respondent’s estimate of HIV prevalence in their affiliated specialty/department – As expressed on a 5-point Likert scale (HIV patients are definitely not seen often – HIV patients are definitely seen often)

Chapter 2 - Attitudes, barriers and facilitators for HIV testing

1. In the past year, I offered patients with [IC] an HIV test [Never – Very often]
2. When I see a patient with [IC], I plan to offer them an HIV test [Completely disagree – Completely agree]
3. In the future, I expect the likelihood that I will offer a patient with [IC] an HIV test [Very unlikely – Very likely]
4. I find offering a patient with [IC] an HIV test [Very unimportant – Very important]
5. I find offering a patient with [IC] an HIV test [Very negative – Very positive]
6. I find offering a patient with [IC] an HIV test [Very uncomfortable – Very comfortable]
7. I find I am [Very incapable – Very capable] of offering a patient with [IC] an HIV test
8. I find offering a patient with [IC] an HIV test [Very difficult – Very easy]
9. My colleagues find offering a patient with [IC] an HIV test [Very unimportant – Very important]
10. Patients with [IC] [Definitely do not – Definitely do] expect me to offer them an HIV test
11. Within my specialty, HIV testing in patients with [IC] is discussed [Never – Very often]
12. HIV testing in patients with [IC] is recommended in our guidelines [Definitely not – Definitely]
13. Ordering an HIV test for a patient with [IC] is [Very hard to organize – Very easy to organize]
14. Offering a patient with [IC] an HIV test ensures better health outcomes [Definitely not – Definitely]

Chapter 3 - Invitation to a subsequent interview

1. Are you willing to participate in an interview on this topic? If so, please leave your name and contact information below.
