# Supplementary material for: Promoting HIV indicator condition-guided testing in hospital settings (PROTEST 2.0): study protocol for a multicentre interventional study
Source: BMC Infect Dis. 2021 Jun 2;21:519. doi: 10.1186/s12879-021-06183-8 (PMC8173796; doi:10.1186/s12879-021-06183-8)
Supplement: Supplementary file 2 — Additional file 2: Supplementary appendix 2. Semi-structured interview guide. This supplementary appendix provides additional information on the recruitment and enrolment for the semi-structured interviews, as well as the interview guide that will be used in this study. [file 12879_2021_6183_MOESM2_ESM.docx]

**Appendix 2: Semi-structured interview guide**

Recruitment and enrollment

Contact persons from the various specialties at the participating hospitals and respondents of the online questionnaire are invited to participate in a semi-structured interview, which will take place in-person or by telephone/videoconferencing. The interviews will be recorded with the permission of the participant.

Goal

The goal of these interviews is to gain better insight into attitudes, barriers and facilitators for indicator condition (IC)-guided HIV testing in specific specialties and hospitals, in addition to the outcomes from the questionnaire, as we hypothesize that different attitudes and barriers influence HIV testing in different ICs and different opportunities for improvement can be identified. The results of the interviews are used in the educational intervention; identified attitudes, barriers and facilitators are used as guidance during interactive discussion, with the aim of identifying tailored opportunities for improvement.

Semi-structured interview guide

1. Introduction: The interviewing researcher explains the goal of the interview, and the characteristics of the participant (work experience, specialty and hospital of employment)
2. To what extent is HIV testing in patients with this IC a point of attention in your specialty? E.g. is it discussed/checked at multidisciplinary meetings or patient rounds? Is there a standard checklist in use for HIV testing in these patients? Is HIV testing among these patients a quality indicator in your specialty?
3. Is HIV testing in these patients recommended in your local and national specialty guidelines? Why (not)? Do you think your colleagues are aware of this?
4. The proportion of patients with this IC that were tested for HIV within 3 months around IC diagnosis is … [the proportion based on the baseline assessment in this study are presented]. What is your opinion on this proportion? Why do you think it is not 100%?
5. Do you think this proportion needs to be improved?
6. Which opportunities for improvement do you see for your specialty? What would work and what would not work?
